# Understanding mobile application development and implementation for monitoring *Posyandu* data in Indonesia: a 3-year hybrid action study to build “a bridge” from the community to the national scale

**DOI:** 10.1186/s12889-021-11035-w

**Published:** 2021-05-31

**Authors:** Fedri Ruluwedrata Rinawan, Ari Indra Susanti, Indah Amelia, Mulya Nurmansyah Ardisasmita, Widarti, Rima Kusumah Dewi, Dani Ferdian, Wanda Gusdya Purnama, Ayi Purbasari

**Affiliations:** 1 Department of Public Health, Faculty of Medicine, Universitas Padjadjaran, Jl. Eyckman No. 38, Bandung, West Java 40161 Indonesia; 2 Center for Health System Study and Health Workforce Education Innovation, Faculty of Medicine, Universitas Padjadjaran, Jl. Eyckman No. 38, Bandung, West Java 40161 Indonesia; 3 Mother and Child Health Division, Department of Public Health, Faculty of Medicine, Universitas Padjadjaran, Jl. Eyckman No. 38, Bandung, West Java 40161 Indonesia; 4 Biostatistics and Epidemiology Division, Department of Public Health, Faculty of Medicine, Universitas Padjadjaran, Jl. Eyckman No. 38, Bandung, West Java 40161 Indonesia; 5 Midwifery Master Study Program, Faculty of Medicine, Universitas Padjadjaran, Jl. Eyckman No. 38, Bandung, West Java 40161 Indonesia; 6 Puskesmas Sungai Durian, Jl. MT Haryono Gg. Wiyata 2, Kelurahan Kapuas Kanan Hulu, Kecamatan Sintang, Kab. Sintang, West Kalimantan 78614 Indonesia; 7 Makassar Regional General Hospital, Jl. Perintis Kemerdekaan No.KM.14, Daya, Kec. Biringkanaya, Kota Makassar, South Sulawesi 90243 Indonesia; 8 Informatics Engineering Study Program, Faculty of Engineering, Universitas Pasundan, Jl. Dr. Setiabudi No.193, Bandung, West Java 40153 Indonesia

**Keywords:** Action research, Android, Community health workers, Mother, *Posyandu*

## Abstract

**Background:**

Limited information is available on how mobile health (mHealth) application (app) technology on mother and child health (MCH) is developed. This research aimed (a) to explore the process of developing mobile apps for MCH community-based services in the Indonesian setting of *Pos Pelayanan Terpadu* (*Posyandu/*Integrated Health Service Post), (b) to determine the feasibility of using the app by community health workers (CHWs), and (c) to evaluate the scalability of the mobile app at the national level in Indonesia.

**Methods:**

A hybrid method was used to synergistically combine the action research principles and mixed methods comprising qualitative and quantitative methods. This study was conducted in the Pasawahan District, Purwakarta, Indonesia, from 2017 to 2019. Content analysis, coding, and categorizing were performed using NVivo 12 Pro for transcribed data. The Wilcoxon test (2018 and 2019) was conducted using STATA 15 Special Edition.

**Results:**

(1) The use of a CHW notebook for data entry into the *Posyandu* Information System book delayed the data reporting process, resulting in the need to develop a mobile app. (2) There were significant differences in CHWs’ knowledge (*p* = 0.000) and skills (*p* = 0.0097) on training (2018) and *Posyandu* phases (2019). (3) A total of 964 *Posyandu* have been registered in the *Posyandu* mobile app from almost all provinces in Indonesia.

**Conclusions:**

The three-year hybrid approach includes the crucial phases that are necessary to develop a mobile app that is more user-friendly and can act as a substitute for CHWs’ book. Hence, its implementation is promising for use at the national level.

**Supplementary Information:**

The online version contains supplementary material available at 10.1186/s12889-021-11035-w.

## Background

Mobile health (mHealth) applications (apps) in the health sector have been publicly utilized and have effectively improved community health services [1, 2]. However, their implementation may be complicated by utility issues among community health workers (CHWs) or cadres of the mother–child health (MCH) services in the Indonesian setting, also known as *Pos Pelayanan Terpadu* (*Posyandu*) or the Integrated Service Post [2, 3]. Action research with a qualitative approach can be used to explore the feasibility of an Android-based health app [4, 5]. However, recent systematic reviews provide little information on how mHealth apps are designed using a qualitative approach to enhance CHWs’ performance in community-based MCH services [6–10]. In 2019, some literature reviews pointed out the lack of baseline data before evaluation and experimental studies regarding CHWs and mHealth [11].

A qualitative approach in action research is crucial to construct a foundation of evidence for designing an Android-based app. This approach is used to explore the degree of acceptability of the CHWs and mothers for the new intervention which may include challenging technical problems, such as mobile network coverage, internet access, and device maintenance [12]. Understanding the technical aspects of current *Posyandu* activities is essential to improve CHWs’ work. These activities comprise five categories: registration, height and weight measurements, documentation, education, and healthcare (family planning and immunization). Based on the activities offered, *Posyandu* is divided into four levels that are classified by size: *Pratama* (five *Posyandu* activities performed by fewer than five CHWs at irregularly recurring intervals)*, Madya* (activities in *Pratama* + more than eight activities per year)*, Purnama* (activities in *Madya* + independent community funding with < 50% of the participants), and *Mandiri* (activities in *Purnama* + > 50% of the participants). During registration, a national report, called the *Posyandu* Information System (PIS) book, provided by the Ministry of Home Affairs must be completed. The village midwives provide information to CHWs, who then complete the form under the midwives’ supervision. However, this leads to delays because CHWs must find previous records and add the new information for the mothers’ and children’s in the PIS book [3].

Owing to time constraints, many CHWs temporarily enter the mothers’ and children’s names and data in a separate book before rewriting them in the monthly and yearly national report. Then, after all *Posyandu* activities are completed, they input the recorded data in the PIS book. The time-consuming nature of these data entry and reporting processes results in a time-management challenge for CHWs. Therefore, a new intervention must be developed to save CHWs’ time. This can be achieved by exploring their perspectives and habits in using the *Posyandu*. This time-saving intervention is essential because CHWs play a central role in connecting the community and national levels through healthcare providers in *Pusat Kesehatan Masyarakat* (*Puskesmas*) or Public Health Centers spread across the country [13]. CHWs submit a monthly report that conveys the community’s condition to the *Puskesmas’*, and this report serves as a basis for creating a budget for community health programs.

Many mHealth technologies are already available in low- and middle-income countries. Using mHealth can support CHWs in delivering health care services [9, 12, 14], community case management [2, 15], and health behavior interventions [8, 9]. A previous report described the use of mHealth services by midwives collaborating with CHWs [16]. Some studies have noted the use of mHealth to help provide healthcare services for patients and families [7], including for palliative care [17, 18], which further highlights the pivotal role of mHealth [19]. A recent literature review identified that data collection may be the main challenge in many community programs and that using mHealth should be included in the training of CHWs [9] so they can provide more effective support [20]. The World Health Organization (WHO) and United Nations Children’s Fund (UNICEF) recommend that countries should foster the quality of care, monitoring, and assessment using innovative approaches, including maximizing the use of the handheld phone. Systematic data collection, aggregation, analysis, and reporting from the smallest administration area (subnational level) to the national level is crucial [21].

Building the capacity, i.e., knowledge and skills, of CHWs and supporting them in data monitoring and reporting or evaluation within a flexible timeframe is the primary factor in sustaining community health programs [9, 13]. Thus, identifying the key obstacles in its implementation is an continuous process [22]. mHealth is promising because CHWs can quickly learn to use this technology provided that it is adjusted to the local context, such as the language and a user-friendly interface [16]. The monthly report extracted from PIS is required for *Puskesmas* midwives in the implementation of an mHealth app. This report includes nutrition data of infants, toddlers, pregnant women, exclusive breastfeeding mothers in the postpartum period, and couples of reproductive age.

Many strategies can be applied to motivate mothers to come to the *Posyandu*. These strategies include monitoring the nutrition status of infants and toddlers, providing immunization status and pregnancy data, providing nutrition counseling to control MCH programs, and emphasizing the benefits of visiting the *Posyandu* [3, 23]. Data recording and reporting are essential because the stored data can be used as a baseline to create evidence-based strategies and evaluate their effectiveness for the *Posyandu*. Further, such recording and reporting can help the *Puskesmas* and village office allocate the necessary budget for improving the existing preventive healthcare programs and generate feedback for each program’s outcomes. A recent literature review determined that measuring the effectiveness of MCH preventive interventions by CHWs is critical for the MCH programs of a country [20].

However, data recording and reporting by CHWs in the *Posyandu* can be complicated in Indonesia. This is a result of the overwhelming number of services provided by CHWs. For example, CHWs must invite mothers and their children to come to the *Posyandu* and approach those who do not come. Additionally, CHWs must communicate with subvillage and religious leaders through community engagement programs to encourage mothers and children to come to the *Posyandu*. Further, in the *Posyandu*, CHWs must manage the registration, measure the children’s weight and height, maintain a data book, educate the mothers, and refer them and their children to the village midwife for immunization appointments, etc. As a result, data recording and reporting workload must be simplified. Critical problem identification should be conducted as evidence-based initiation and development of solutions. The findings of this study will contribute toward improving the use of mHealth among CHWs and mothers. Hence, this research study aimed (a) to explore the process of developing a mobile app, (b) to perform the assessment of adaptability and feasibility of using the app by CHWs, and (c) to evaluate the potential scale of the *Posyandu* mobile app to the national level in Indonesia.

## Methods

Action research comprises step-by-step development beginning with the identification of initial ideas, implementation, and feedback for improving these ideas. This process is an iterative cycle that continuously supports better action or intervention. In this case, the assessment outcomes should meet the community’s needs to create the necessary implementation/intervention, which will help the CHWs create a *Posyandu* data report. The information is vital to develop a plan and design an intervention starting from the initial blueprint. The intervention design as well as further actions to deliver the intervention should be presented to the community to allow further improvement. The ideas and intervention must be prepared and advocated to the relevant stakeholders, e.g., government and private sectors.

In general, the combination of action research principles and mixed-method research (qualitative and quantitative methods), henceforth referred to as a hybrid approach, was synergistically applied for end users. The core design comprises complex mixed-method designs, which incorporate more designs into sequences [24]. This hybrid approach was deployed in the following eight phases: (1) determining issues by analyzing and understanding users’ activities, which refers to the process of exploring their activities at the *Posyandu;* (2) making a design prototype blueprint; (3) evaluating the blueprint with the users through presentations and discussions in which steps 2 and 3 may be repeated in case of any feedback; (4) designing the prototype; (5) creating a dynamic design prototype or a dynamic design that is programmed with planned features but still not in the executed form; (6) evaluating the design with the users to prevent any miscommunication; (7) testing the executed prototype; and (8) implementing the final version of the graphical user interface (GUI). GUI designing ensures that a good user interface program is complemented by good quality. It spots interactions between the user and the mobile app using graphical information or visual widgets, such as text boxes and clickable buttons. Such an approach is necessary to bridge the quality and users’ needs so that it can be accessed easily by users [25].

**Fig. 1:**
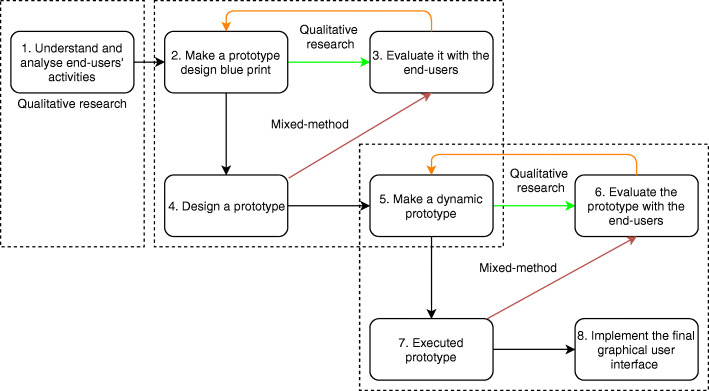
Hybrid approach (action research principal and mixed-method research design) to develop the *Posyandu* application

The initial part of the action research was conducted using a qualitative approach because community insight must be evaluated after collecting information. Thus, the GUI can fulfill the needs of the community. Based on phases 1–2–3-2-4, a qualitative design made in 2017 was used to create, evaluate, and improve a blueprint and then design a prototype. In 2018, a sequential exploratory mixed-method design and an embedded qualitative approach were performed using phases 4–3–2-4-5 to evaluate and improve the blueprint and prototype. After the qualitative approach, the knowledge and skills of the CHWs in using the mobile app were checked using a quantitative approach during training. Their feedback was also incorporated into this approach, which resulted in a dynamic prototype in 2018. Then in 2019, using phases 5–6–5-7-8, the next qualitative and embedded quantitative–qualitative designs were tested to identify the end users’ knowledge and skills (quantitative), including feedback from CHWs on the GUI of the mobile app. Feedback from village midwives who supervised the CHWs was also obtained (Fig. 1).

In practice, prototyping was performed using the spiral model development process. The eight spiral or prototyping phases resulted in incremental refinement of the app through each iteration. A user-centered design was implemented when starting the development from low to high fidelity persona. The stage cycle was iterative, wherein the context was identified directly from the users. This was explained in technical terms, and the programmer used these as a reference for improvement. The development of a low fidelity persona began with a qualitative study to create a blueprint. A focus group discussion (FGD) for the CHWs and mothers was organized to discuss their activities in the *Posyandu*, the importance of mHealth, and the mobile app’s main features (box 1 in Fig. 1). The first iterative cycle began with the first version of the blueprint (box 2 in Fig. 1) created on the basis of the FGD, and it was presented again during the FGD using a projector and PowerPoint slides as a paper prototype. The discussion produced feedback (box 3 in Fig. 1) regarding the main features such as registering and logging in, reports, data input mechanism, and alerts (Supplemental Table 1), followed by approval of the improved features of the second version of the blueprint (box 2, iterative) before developing the first high fidelity persona in the Android package (APK) form (box 4, Fig. 1). The CHWs accepted this APK blueprint as it could help them monitor infants, toddlers, and mothers. They pointed out, *“With the Posyandu mobile application, it is possible to check the development of children, examine pregnant mothers, immunization.”* The mothers also accepted it because they could use it to monitor their toddler’s development. They said, *“(We) need to know our child development so that we can monitor our child by ourselves”* (Supplemental Table 1).

The first APK was developed and then shared with the CHWs and mothers via the SHAREit app. They then installed it as a trial version and used it in training. An FGD was organized to discuss the trial version in training (box 3, iterative). Feedback on the toddler data input and display, the app menu, and the online report was used to improve the trial version (boxes 2 to 4, iterative) to create a more dynamic solution prototype (box 5, Fig. 1). Additionally, the CHWs and mothers provided feedback about the learning process and the app guidebook (Supplemental Table 2). The dynamic prototype could store its data into a server and help the CHWs’ in the *Posyandu*. This prototype served as the starting point for the next iterative cycle.

The prototype was then re-evaluated in a FGD. This prototype was dynamic in its changes and improvements, depending on the iterative cycle process that was based on the users’ feedback (box 5–6-5). This feedback included improvements in identity, account, and web creation in the future (Supplemental Table 3). For example, if users forgot their password, they can contact the admin via WhatsApp to reset their password and obtain an alternative password. This feedback was provided by a midwife who stated “*Oh, I forgot the password. It is not possible to login. It would be great if an alternative password exists*.” Then, once the users approved the dynamic prototype, it was then executed and launched in Google Play (GP) (box 7). The dynamic prototype was tested by end users mainly in the research area. A research published in 2016 pointed out that internet mobile phones, including phones using the Android platform, are used by at least 78% of the Indonesian people. In 2019, the Indonesia National Broadband Plan (*Rencana Pitalebar Indonesia*) targeted a mobile broadband penetration of 100% in urban areas and 52% in rural areas [26]. The increasing number of people in the country using the Android platform is why this platform with GP was selected as the native mobile app.

The executed prototype was not the final version, even though it had already been launched on GP. It was an unreleased version and was limited to 6000 downloads on GP. Before confirming the final GUI (box 8) to release the final version, the iterative cycle was carefully applied for further development. During iterative software development process, feedback was taken from comments on GP that was then used to refine the mobile app. Additionally, we received information about crash events and device compatibility, which was used to support development. We also conducted observations and another FGD for CHWs using the app’s unreleased version when running the *Posyandu*. We considered the feedback from this FGD as a basis to develop the final GUI of the app (box 8 of Fig. 1). However, the mobile app still requires development to accommodate the users’ needs in the future.

**Fig. 2:**
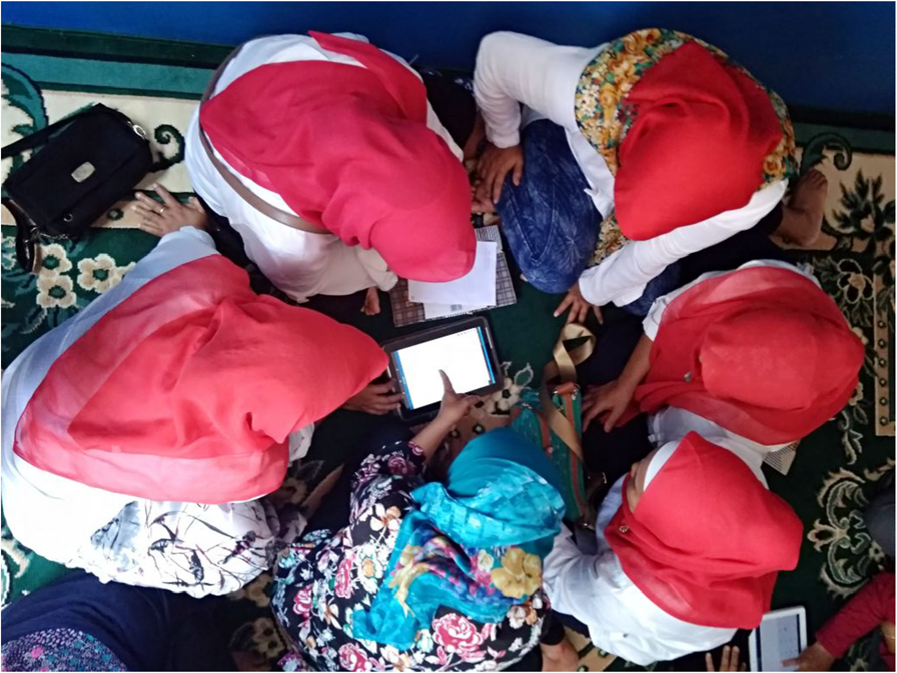
The focus group discussion process for a dynamic evaluation and execution of the *Posyandu* application. This figure was created by the authors

The qualitative portion of the research was conducted by FGDs consisting of 10–14 participants (Table 1), which included CHWs and mothers in 2017, CHWs in 2018, and CHWs and midwives from every village in the Pasawahan District in 2019. FGDs were conducted because these people were engaged more at the *Posyandu* and were able to use the mobile apps. Midwives play a role as *Posyandu* supervisors in each village and were thus included in these FGDs. The research was conducted in Pasawahan District, Purwakarta Regency, West Java, Indonesia. FGD participants were interviewed with open questions about the challenges in running the *Posyandu* to understand their concerns and propose adequate solutions. Then, the input was considered and the users’ feedback was reviewed after solution implementation. A qualitative sample was chosen using a purposive sampling technique according to the participants’ activity and ability to use a smartphone. The illustration for this explanation is provided in Fig. 2.

**Table 1:** Number of Participants in Each Phase of the Study

Participants	Number of participants in each phase year				
	2017	2018		2019	
	Qualitative	Qualitative	Quantitative	Qualitative	Quantitative
CHWs	13	12	171	10	156
Mothers	14				
Midwives				11	

*CHW* community health worker

An instruction/user training guide was required to enable the cadres to operate the *Posyandu* mobile app. Qualitative data were acquired through FGDs with the *Posyandu* cadres to obtain their opinions on the app usage instructions. Twelve *Posyandu* cadres attended the FGD representing each village in the Pasawahan District. The information acquired was used to establish a user guide, which was then delivered to the cadres to use as a reference during training.

Then, quantitative data were collected to assess the cadres’ knowledge and skills in using the *Posyandu* mobile app during training. The knowledge tested included account registration management, benefit of the app, records of pregnant women and toddlers and their physical examination results, and PIS. The skills tested included account registration; app login; data entry, data display, data search, examination data entry, examination data display for infants and toddlers; data entry, data display, examination data search for pregnant women; and logging out. Knowledge was assessed using questionnaires whereas skills were assessed through quantitative observation using a checklist (Supplemental Tables 5 and 6). During the observation process, the researchers were assisted by 10 selected facilitators who were the most active and trained cadres.

The facilitators were trained to use the *Posyandu* mobile app according to the instruction book. Each facilitator was able to operate the *Posyandu* mobile app and guide other cadres on how to use the app. Each facilitator was provided with an Android mobile phone/tablet and was in charge of 8–10 cadres. The facilitators organized a visit schedule for the cadres they were guiding. For a month, the *Posyandu* cadres were guided by the app trainer by using the provided mobile phone/tablet in turns.

The quantitative research sample size was calculated with the objective of assessing the significance between two time points (training time and implementation time). We used the per group sample equation from Hulley SB et al. (2007) with an α of 0.05 (two-tailed hypothesis), a β of 0.10, and an effect size from previous research of 0.56 [27, 28], resulting in 72–86 required respondents [29]. The treatment group comprised cadres who met the inclusion criteria (active cadres) and who participated in the 1-day training with an instruction book and were guided by a trained cadre facilitator*.* The control group comprised cadres who met the inclusion criteria and only participated in the 1-day training. Knowledge and skills were assessed 1 month after training (2018) and during the implementation of the *Posyandu* (2019).

The app was released on GP in December 2018 to scale up the local users’ impact at the national level. The distribution of registered *Posyandu* on the mobile app in Indonesia was analyzed. An Excel file comprising all *Posyandu* that had registered with the mobile app was downloaded from the app administrator website, and the data were registered in a database server. The data quality was checked using STATA version 15.1 Special Edition License (StataCorp LLC, Texas, USA). Then, a distribution map of the registered *Posyandu* up to December 31, 2019 was created using the QGIS version 2.6 (open source) shapefile of the 34 provinces in Indonesia.

### Analysis

In the qualitative analysis, the responses of the mothers, CHWs, and midwives were coded and categorized for end-user activities, needs of the mobile app, main features of the app (2017), toddler data input, display, *Posyandu* mobile app components, benefits of the *Posyandu*, obstacles in using the *Posyandu* mobile app, learning process, guidebook, its information, cadres expectations and worries (2018), the app development on identity, account, the needs of the website, new menu, and the advantages and disadvantages of the app (2019). Similar answers were categorized into nodes/codes, and grouping’s insights were used to name the node. This step was intended to build and understand the critical connection between the needs and recommendations, which were to be used as feedback for designing the mHealth app. The content analysis nodes in NVivo 12 Pro License were used in the analysis, and the context of the diagram and entity relationship diagram were extracted from the program. Subsequently, the results were exported and could be displayed as a report.

Quantitative data analysis conducted using STATA version 15.1 Special Edition License was used to observe the characteristics of treatment and control respondents. Their knowledge and skill scores in 2018 (training) and 2019 (*Posyandu* activities) were compared using the Wilcoxon signed-rank test because the data were not normally distributed (Shapiro–Wilk normality test < 0.05). The effect was also analyzed by observing the Z-score (standardized test statistic, produced by STATA) divided by √N (*N* = number of all respondents) and time difference (training time, 2018 and implementation time, 2019) [30].

## Results

### Data flow diagram result in the database

#### Scope of the system

**Fig. 3:**
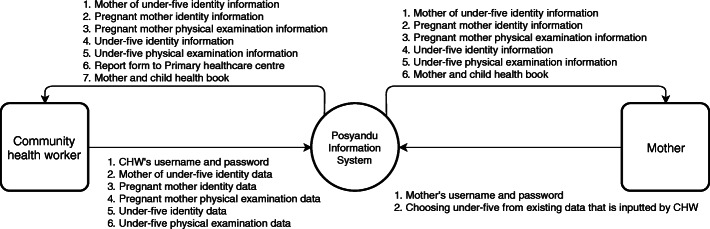
Data flow diagram of the mobile health application design in the *Posyandu* Information System

PIS was developed to support *Posyandu* data management and analysis. The collected data were recorded by CHWs, including usernames and passwords, mothers’ records, pregnant women’s records, pregnant women’s physical examination results, toddlers’ records, and toddlers’ physical examination results. CHWs can also access this information in the monthly report section of the app. The form was already categorized into a monthly and yearly national form. Parents could view the information about their toddlers by performing the following steps: registering using their username and password, logging in with the registered credentials, and selecting their children’s records that were already listed by the CHWs. Parents can access information on their records, physical examination results (of a pregnant woman), their children’s records and physical examination results, and the MCH book. This information is depicted in Fig. 3.

### Initial phase qualitative research results (2017)

The first result comprised the qualitative part of the research. Input collected from the CHWs and mothers were combined in one table and divided into themes, key insights, and quotes. Table 2 and Supplemental Table 1 show the main problems faced by the CHWs, a disorganized yearly data record and report. They stated that the data were hand-written in their notebooks and this was easier for them than to write it immediately in the big book report or the PIS book. One CHW confirmed that *“the paper notebook can be used immediately.”* Another CHW ascertained that *“if the data is written directly to the PIS as the mothers come, it will blow my head (since it is) confusing.”* The CHWs did not have time to put the children’s names in an orderly manner, as pointed out by one of the CHWs. Then, there was also the issue of delay in reporting to the *Puskesmas*. This delay was a result of the double work of data entry to their personal book and report entry to the big book. The CHWs felt that this work was redundant. A CHW stated in the FGD that *“… we have to write the names to the Posyandu Information System book in an orderly manner.”* They indicated that they required a solution such as mHealth to facilitate the data recording and reporting process. The CHWs described it as *“(something) like a tool, but it can be reaccessed, like an archive. Because we need it when Puskesmas requests (a report), sometimes it can be reaccessed.”*

**Table 2:** End-User Activities, the Needs of Mobile App, and Main Features

No	Theme	Key insight
1	End-user activities	
	a. CHWs’ activities	i. The direct use of the CHWs’ notebooks for MCH service ii. Delay in transcribing the information into the *Posyandu* information system (PIS) book iii. The PIS book is extremely difficult to implement
	b. Mothers’ activities	i. Mothers who work will ask others to bring their children to the *Posyandu*: - babysitter - grandmother - neighbor
2	The needs of the mobile app	
	a. CHW	i. Monitoring infants, toddlers, and mothers ii. CHWs need a mobile app to report to *Puskesmas*
	b. Mother	i. Mothers need a mobile app to monitor their toddlers
3	Main features	i. Login ii. 12-Month Reporting Format iii. Similar to the reporting form used in the *Puskesmas* iv. Automation of infant data input when the app is re-opened v. Child growth graph vi. Automatic alert of child growth

*CHW* community health worker, *MCH* mother and child health

As Supplemental Table 1 presents, the app would help mothers supervise their toddlers’ growth and development. The mothers stated that *“(We) need to know our child’s development so that we can monitor them by ourselves.”* Moreover, working mothers need a way to monitor their children’s growth when their family members or neighbors are in charge of taking them to the *Posyandu*. One mother said, *“For example, this (child), the child is taken care of by another person (because) the mother is working.”* They hoped for a way to avoid repeatedly asking the CHWs about their children’s growth because *“(it was) just not practical.”* It was revealed in the FGDs that mothers need the *Posyandu* mHealth app “*so that (they) can access it privately (and immediately). Thus, (they) do not have to ask the CHWs continuously.”*

The activities, including the quoted difficulties above, served as input to extend the context in the app’s blueprint. Moreover, other inputs, such as the registration, the connection between mothers’ and children’s data, and data entry, can also have automatically reported outputs such as governmental forms, child growth graphs, and automatic alerts on the child’s growth status. These main features are depicted in Supplemental Table 1.

**Fig. 4:**
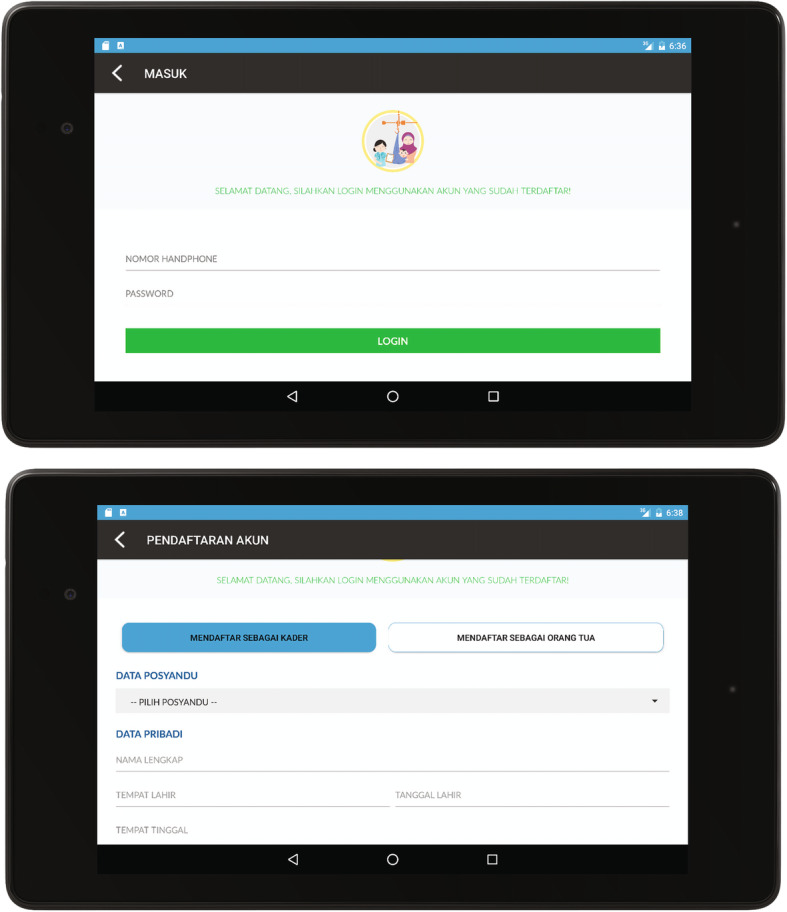
Log in page and registration page of the *Posyandu* mobile health application

Figure 4 illustrates the app’s initial phase for the CHWs/cadres and mothers using touchscreen smartphones. Initially, the registration menu in the app was different for CHWs and mothers. As one of the respondents stated, *“First, we click on the Posyandu app, then we register in it, after that we click it once more, then we are connected to our children’s data.”* Mainly, personal data and the name of the nearest *Posyandu* were necessary when registering before they could log in according to their role as a cadre or mother.

### Middle phase qualitative and quantitative research results (2018)

Table 3 and Supplemental Table 2 illustrate the qualitative theme and key insights that emerged during the CHWs’ training for using the *Posyandu* mHealth app in 2018. The cadres recommended that the notification feature for monthly weight data should be automated. Height was measured according to government and WHO guidelines. However, they confirmed that *“We do not measure the height monthly but only once every several months.”* After the cadres input the required data, they wanted to check whether the toddler growth chart was updating automatically. Additionally, they expected the information to be available anytime. To quote, *“We want it to be like … online reporting, so we do not need to measure the number of decreases”* (Supplemental Table 2). By this, they expected the app to ease their duties in the *Posyandu* by recording the data and simultaneously submitting the report directly to the *Puskesmas*. Nonetheless, when it came to using the app in the *Posyandu*, they still felt that some obstacles remained. For example, *“A while ago, some data was successfully stored, but some were unsuccessful.”* They believed that the mHealth app would not be supportive when the *Posyandu* was crowded: *“During the Posyandu working day, it will remain crowded so that the data entry will be done after the end.”* They were also worried about internet availability when they ran out of funds.

**Table 3:** The use of Posyandu mHealth Application by CHW

No	Theme	Key Insight
1	Toddler data input	a. Toddler body weight measurement
		b. Toddler body height measurement time
		c. Toddler body height measurement according to the WHO
2	Toddler data display	a. Toddler data can be accessed anytime
		b. Toddler measurement result display
		c. Parents can access toddler data
3	*Posyandu* mobile app components	a. *Posyandu* mobile app menu
		b. Online report
4	Benefits of *Posyandu* mobile app	a. *Posyandu* reporting and recording is easier than using the big book for reporting
		b. Facilitate cadres’ duties in the *Posyandu*
5	Obstacles in using the *Posyandu* mobile app	a. Confused/need to adapt
		b. Unsupportive *Posyandu* situation
6	Learning process	a. Cadres’ knowledge of the *Posyandu* mobile app
		b. Cadres’ skills for using the *Posyandu* mobile app
		c. Cadres need more training with the app
7	*Posyandu* mobile app guidebook	a. Significance of the *Posyandu* mobile app guidebook
		b. Guidebook format
		c. Guidebook size
		d. Guidebook writing style
		e. Images in the guidebook
8	Information in the guidebook	a. Instruction
		b. How to register an account/log in
		c. How to input toddler data
		d. How to input pregnant mother data
9	Cadres’ expectations	a. Tablet/mobile phone provision
		b. Use of the app in the *Posyandu*
10	Cadres’ worries	a. Internet quota availability

The cadres still believed that the app could be of great assistance to them after considering the advantage and disadvantages. The learning process played a central role in this belief. During the training, they stated, *“We think we can use it because we are used to using and playing with a mobile phone. However, before that, the application should be made available first* (on GP)*.”* The app was available in the APK form during the training, and it had not been published on GP at the time. In late 2018, it was released on GP to make it more widely available and accessible.

During the training process, they required more written information in the form of a guidebook. The cadres also coordinated with village officers regarding any app-related issues. One such issue was internet usage: *“… the Posyandu does not have any budget (to cover it). I asked the villagers about the internet quota fee, and they shook their heads.”* Regardless, it was expected that the cadres could use the app and put it into practice at the *Posyandu* after the training was completed. They also expressed their interest in using the app. *“If using the application if possible, then so be it, (I) cannot wait to use it.”* More information regarding their feedback is shown in Supplemental Table 2.

### Final phase qualitative and quantitative research results (2019)

Tables 4 and 5 and Supplemental Tables 3 and 4 present the conclusion of the FGD with cadres and village midwives for the app development and the advantages and disadvantages of the *Posyandu* mHealth app, which incorporated the ideas from other FGDs of cadres and midwives. When using the app during *Posyandu* activities, some corrections were required, as noted by one of the informants: *“Here, the name of my village in this application is wrong.”* Other feedback recommended inserting a photo in the account information and an alternative password. As a cadre supervisor in several *Posyandus*, one of the midwives suggested that *“… in the future, it would be great if there is an access for the village midwife and not only for the cadres,”* which would positively affect future app development. The creation of a website was also discussed for reporting purposes. The midwives perceived that reporting using a laptop would be easier than with a mobile phone. More feedback is presented in Table 4/Supplemental Table 3.

**Table 4:** Cadres’ and Village Midwives’ FGD Results on *Posyandu* Mobile App Development

No.	Theme	Key Insight
1.	Identity	Village name correction in the editing menu
2.	Account	Account owner photo
		Alternative password
		Individual account for village midwife
3.	Website	Web creation
4.	New menu	Pregnancy age automatic calculation

**Table 5:** Advantages and Disadvantages of the Posyandu Mobile Health Application

No	Analysis Result	Advantages	Disadvantages
1	User		
	a. Receipt	Cadres’ approval of the application	
	b. Problem Solving	Problem solution by the cadres when facing difficulties	
	c. Skill	Cadres’ skills in operating the *Posyandu* mobile app	
	d. Resistance		Time-consuming because there is a reluctance on the part of some cadres to change to digital system-based services
2	Organization		
	a. Policy	Leadership of a village midwife as the direct supervisor of *Posyandu* activities	
	b. Organizational support		Cadres’ facility in implementing the *Posyandu* mobile app is still necessary
	c. Standard operating procedure (SOP)	Positive response in the advocacy of the government	Double work burden of manual and digital tasks because no SOP regulates the implementation of the *Posyandu* mobile app
3	Technology		
	a. Application/*Software*		
	i. Accuracy	Appropriate and correct use of the application	
	ii. Facility	User-friendliness of the application when being operated by the cadres	
	iii. Availability	Availability of the application in Google Play to be used or operated	
	iv. Relevance	Conformity of the application menu with the needs of the cadres or as planned by the government	
	v. Punctuality	Real-time condition of the application to display the information or examination result	In regions with low network coverage, an offline version of the application is necessary for data entry, which will be submitted after the network appears
	vi. Challenge		Application bugs must be fixed
	b. *Hardware*		
	i. Network		Unstable network for some providers/carriers
	ii. Mobile Phone		Some versions of Android are not compatible with the application
	iii. Quota		Some cadres do not have an internet quota
4.	Environment		The situation that is not conducive (queueing issue) during the *Posyandu*’s business time

As indicated in Table 5 and Supplemental Table 4, the advantages and disadvantages of implementing this app were analyzed from the perspectives of the user, organization, technology, and environment.

Table 5 and Supplemental Table 4 illustrate some cadres’ resistance in changing their habits from using paper-based services to digital services. In practice, village midwives assisted in supervising and encouraging the app’s implementation during *Posyandu* activities. Continuous organizational support from the village was vital in 2019, which was also applicable in the previous year. A standard operating procedure (SOP) issued by the government is essential. This statement should address the village office’s leadership, district, *Puskesmas*, and district health office (DHO). The SOP would strengthen the app’s implementation despite the burden of double work burden initially; this burden would disappear once the users get accustomed to it.

Technology is at the core of this implementation; thus, software and hardware analyses are crucial. In 2018, the app was launched on GP, and in 2019, it was available for downloading and operating on Android mobile phones. The app answered the users’ needs that were identified in the previous years of action research, such as real-time data entry and reporting. However, in regions with low network coverage, an offline version was still necessary for app development. Thus, data could be submitted when network was available. While the issue of unstable network was noted in the areas of low network coverage, some mobile phones were not compatible with the app version. The app version must be continuously improved to make it more compatible with all mobile phones.

The cadres’ knowledge and skills in implementing the *Posyandu* mobile app during the training (2018) and *Posyandu* activities (2019) were evaluated as ongoing research. The respondent characteristics are presented in Table 6. Most of the respondents were aged > 35 years, and most of them had received secondary education or attended junior high school. In 2018, the respondents comprised 171 *Posyandu* cadres. In 2019, 8.77% of the respondents were no longer reachable or could not be followed up (*n* = 15). The remainder of the respondents included 79 and 77 people in the treatment and control groups, respectively. Hence, a total of 156 respondents were assessed.

**Table 6:** Respondent Characteristics in Pasawahan Sub-District, Purwakarta District in 2018 and 2019

Characteristics	2018		2019	
	Treatment (*n* = 86)	Control (*n* = 85)	Treatment (*n* = 79)	Control (*n* = 77)
Age (years)				
26–35	18	22	16	19
36–45	32	33	31	33
46–55	36	30	32	25
Education				
Elementary School	24	31	21	26
Junior High School	32	33	30	30
Senior High School	30	21	28	21

The comparison between the participants’ knowledge during the training (2018) and during app implementation (2019) is shown in Table 7.

**Table 7:** Comparison of Knowledge and Skills during Training and Posyandu Activities

Variable	Occasion	Value				Effect
		Min	Max	Mean	*P*-Value	
Knowledge	Training	84	100	94.69	^a^0.0000	0.34
	*Posyandu* Activity	76	100	91.91		
Skill	Training	7.69	100	85.63	^a^0.0097	0.21
	*Posyandu* Activity	27.63	100	93.05		

^a^Wilcoxon signed-rank test

Based on Table 7, the cadres’ average knowledge and skill scores during training and *Posyandu* activities differed significantly (*p* < 0.05). Knowledge and skills had effect sizes of 0.34 and 0.21, which are considered small and medium, respectively, according to Cohen [30, 31].

**Fig. 5:**
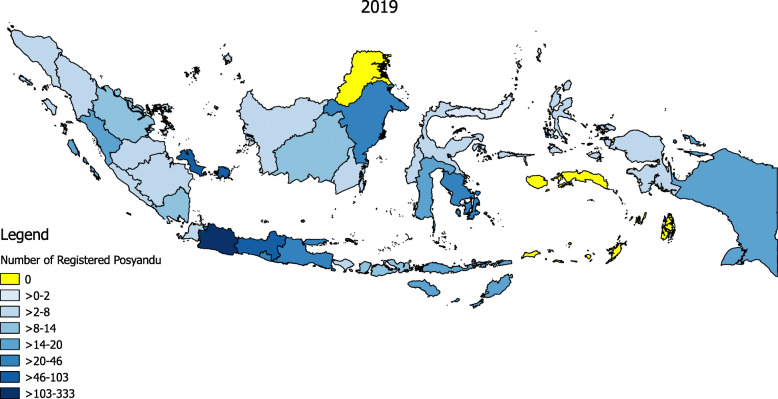
Distribution of registered *Posyandu* in the mobile app in Indonesia until December 31, 2019. This figure was created by F.R.R using QGIS version 2.6 (open source)

Figure 5 illustrates distribution across the 34 provinces of Indonesia until December 31, 2019. As many as 964 *Posyandus* were registered on the mobile app from almost all provinces of Indonesia. The highest proportion of *Posyandus* (34.54%) was recorded in the research area of West Java. Other provinces also showed interest in registering their *Posyandus*. Belitung had the second highest number of registrations, followed by Jakarta, Central Java, and Yogyakarta. There were no registrations from North Kalimantan or Maluku.

## Discussion

Qualitative research is a critical part of action research that is necessary to create a foundation of knowledge. From this foundation, interventions can be developed that solve community problems and adjusts to their knowledge and skills [32]. Some research begins by formulating a direct intervention based on their initiative without first determining the targeted community’s mindset [33]. This practice could prove to be a potential bias when performing the intervention. This can be in the form of a knowledge bias in terms of a gap between the intervention maker or expert-driven method and the end-user of the intervention [33, 34]. Creating mHealth interventions should begin with a theory-driven process followed by taking feedback from the end-user or targeted community [35]. The intervention designer should then determine the details of the design and intervention based on the end users’ feedback [36]. Previous studies have compared different apps that use the top-down and bottom-up approaches and revealed that a bottom-up app was more effective for the community [33]. The hybrid approach (Fig. 1) comprises a complex mixed-method design that begins with qualitative research and is followed by sequential and embedded qualitative to quantitative designs. Incorporating action research into the complex mixed-method design is in a sense a cyclical improvement. For each cycle of action research, the qualitative results are followed by quantitative design; for example, quantifying the significance of the implementation effect will render it more efficient and useful for the next improvement (action research). This effect can be accompanied by qualitative research embedded with quantitative research to clarify the reasons underlying the quantitative research results, which can provide further data and context to the research. The next cycle can thus be more complex as per the problem [37]. This method of a continuous cycle can lead to continual improvement.

There are two types of principal process models in software development. Firstly, there is the traditional linear sequential model of one phase that comprises analysis, design, construction, and implementation. Discussions between software developers and end users only happen at the beginning and end of the process (analysis and implementation). Second, there is the iterative process model, which splits the phase-development process into small iterations [38]. These small iterations comprise more detailed improvements for the app, and the iterations can be combined with qualitative and quantitative research cycle to identify the users’ needs [36]. The second model is slower than the first but fits with developing communities that are also slower in adapting to technology. Our 3-year research study was slower than some studies that also deploy users’ role as an essential factor in software application [39]. One year is insufficient to identify more profound evidence that can help build a bridge to connect users with technology in this hybrid approach research. The present research also seeks potential use on the national scale by preparing technology that already includes this bridge. Technology that is fast but does not have this bridge is essentially ineffective [40]. A lesson from this study is that software developers should provide a bridge for slower communities rather than providing quicker advanced technologies because such communities can barely keep up with those changes. This gives them more time to grow with the development speed of the slower technology. This concept corroborates psychosocial intervention development and implementation, wherein the design process is based on users’ information [40]. This design can provide the connection to fill the gaps between technology and user in accepting that technology. These gaps, including poor internet connection, have been recently discussed [10].

Currently, in Indonesia, the use of Android phones is growing rapidly. A total of 78% of the population used Android phones in 2016, and this rate is predicted to include 100% of the population in urban areas and 52% in rural areas in 2019 [26]. The trend of using short messaging service (SMS) has shifted to the use of internet-based apps for messaging, such as WhatsApp, Line, and Telegram [41]. More apps with higher functionality are also being developed for particular purposes [42], such as data documenting and reporting. One such example is the *Posyandu* app. For certain regions with additional gaps such as low connectivity, it can be very difficult to access the app’s updates and synchronize it to the server. Instead of developing an SMS version, an offline version was developed to overcome the shortcoming of low connectivity in some areas while the online version for documenting more detailed and complex *Posyandu* data was underway. The integration of apps for people in areas with available networks is easier than in areas with no network. The offline version was planned to have the capacity to store data in the Android device and then to synchronize with the server when connectivity is available. For example, when CHWs go to the village office in their area, there is a greater possibility of connecting with the internet using Wi-Fi from the office. Furthermore, internet payment is allocated in the village office budget and is not self-funded by the CHW. A literature review discussed the lack of attention to technological integration and the smaller number of action research studies compared with other software development studies [43]. The use of action research in software development is encouraged. The results of this research may enrich previous studies that employ action research in connecting technology to people.

Mobile app development was chosen as it covers more functions than SMS, which has limited functionality [44, 45]. SMS can be used and is feasible for documenting aggregate data, e.g., clinical case numbers and treatment numbers at a village level aggregate [44], including communication with limited SMS characters [8, 10]. However, sending complex data such as the *Posyandu* data in individual SMS messages would be more expensive and complicated. Furthermore, the effectiveness of using SMS is debatable according to a previous literature review [46].

The application interface in 2017 was considered satisfactory because it was established and displayed based on the community feedback, and culturally embedded factors were explored [47]. In the context of this research, the *Posyandu* cadres and mothers are related in the data flow diagram (DFD), which is the “kitchen” or the back-end of this app. The DFD describes what the cadres and mothers do and what they receive from this app. According to previous research, building an app based on its candidate users’ feedback will juxtapose the user’s local context usage perception gap to the designer to support the community’s adaptation and acceptance [48]. The mobile app technology design can provide more benefits in establishing strong partnerships between stakeholders to leverage the community capacity and empowerment, e.g., the CHWs and mothers [49, 50]. Awareness of the community in terms of preventive healthcare interventions requires capacity building to maintain the CHWs’ and mothers’ knowledge and skills to perform community screening [51]. Previous research in 2018 also stated the needs of a learning process (Table 2). Touchscreen smartphones were used in this research as they support the learning process. These findings corroborate previous recent literature reviews that state that it is better to use touchscreen mobile phones because of their ease of use and minimal need for technical support [12]. However, after training, follow-up showed a medium effect on knowledge and a small effect on skills. This result differs from another study that employed training interventions for CHWs using a module in reproductive health and tuberculosis fields, which demonstrated a larger effect. Although the fields are different, the idea of emphasizing the confidence and satisfaction of CHWs proposed by the research remains essential and relevant [52]. Training should be improved by emphasizing such insights and considering different measures to reach a larger effect in 6–12 months [53]. An excellent impact would be demonstrated by the CHWs engaging more in the intervention and benefiting society and the government [54].

Although the cadres’ knowledge and skills in the *Posyandu* mobile app implementation generally met expectations, the implementation of a new information system is not easy due to the many influencing factors that must be considered. The first factor is the user. Implementing a new information system will be successful if each user has a similar performance expectation that the *Posyandu* mobile app can ease their workload. Performance expectation is a strong predictor of information system utilization interest [55–57]. Another factor is the usage facility, which is defined as when an individual is certain that using the system does not require extra effort [58, 59].

The cadres had more freedom in learning how to use the *Posyandu* app because they utilized it through a personal smartphone, which gave them more time to learn how to operate it. There is ample opportunity for Indonesian citizens, including cadres, to learn using their smartphones because as per research published in 2016, at least 78% of Indonesians had smartphones. The increase in the cadres’ skills was probably because the cadres could learn independently. Although the cadres initially received only some information and had to learn to operate the app independently, they had similar knowledge and skills with the cadres group that received specific training. This independent learning is in line with a previous South African research in 2018, wherein an explorative study demonstrated that the respondent assessment value dramatically increased. However, no intervention was given in that study. The interview results showed that the cadres often gathered and created larger study groups to learn together [52]. In future action research, quantitative research can objectively evaluate video education that can be embedded as part of the mobile app. This research can also be continued by monitoring the steps to identify cost-effectiveness development to strengthen a strong partnership in advocacy programs to different stakeholders [60].

In the Indonesian health system, access to screening in *Posyandu,* which is performed and documented using the *Posyandu* mHealth app by trained CHWs, can substantially help the government improve data management and consequently, the quality of information. The village midwife, nutrition, and health promotion staffs of *Puskesmas* have a role in assisting with the activities of the cadres, including validating the *Posyandu* data before reporting it to the *Puskesmas* [61]; for example, in the integration with government programs for establishing the conceptual framework of stunting reduction interventions. There are five pillars of interventions: (1) commitment and vision of leadership; (2) national campaigns and behavior change; (3) convergence of central, regional, and village programs; (4) food and nutrition security; and (5) monitoring and evaluation. In the fifth pillar, the stunting reduction intervention using the data management system requires effort to bridge the data management at the village to the regency/city level and up to the national level [62]. An example of an app available at the national level is the Integrated Nutrition information system (Integrated Nutrition). The Integrated Nutrition data collection starts with the weight and height data that is obtained every month at the *Posyandu* and is recorded in the register book. Data entry to the information system falls under the responsibility of the *Puskesmas*, which can be performed at the *Posyandu* level as a source of growth monitoring data [63]. However, the implementation of data entry in the Integrated Nutrition app in the research area was still conducted by *Puskesma*s staff based on the results of the measurements reported by the health cadres in *Posyandu*. The cadres’ manual work can create problems, such as delay in inputting data due to the high workload of the *Puskesmas* staff. Therefore, an app can solve this problem [9]. The *Posyandu* app can bridge the problems mentioned above through a data input process performed directly by the health cadres during the *Posyandu*’s working hours. The data can then be directly downloaded, verified, and uploaded by the *Puskesmas* following the format for the Integrated Nutrition app. In this manner, the reporting process can run promptly and can be used as a material for the decision-making process related to reducing stunting.

The advantages of using mHealth for cadres are supported by this research and recent literature reviews [7, 9–12, 46, 60]. Studies that have evaluated the program results found evidence that mHealth assisted CHWs in enhancing the provided treatment quality, services efficiency, and program monitoring capacity [6]. Similar research also revealed that mHealth is considered beneficial for CHWs because it can help them with their duties, support clinical decisions, and send instant data and feedback on performance [2]. Another finding indicates that mobile-based data collection increased the data collection punctuality, decreased error level, and enhanced data completeness [9].

Nevertheless, this research also had disadvantages, such as user resistance, low organizational support, lack of standard operating procedures, low network coverage punctuality, bugs, hardware challenges, and a non-conducive environment (Table 5). Both the advantages and disadvantages can help health promoters plan continuous improvements in mHealth interventions [64]. In general, the CHWs’ role in mobile technology is to collect field-based health data, give warnings and reminders on routine *Posyandu* activities, facilitate health education sessions, and conduct person-to-person communication with parents. A programmed effort from the cadres can strengthen health services performance [54], which focuses on community-based MCH management for primary and secondary health prevention. This research is similar to another study about community case management for children’s illnesses [15] but differs in some task settings. The *Posyandu* CHWs in this research mainly educate healthy people and refer them to the village midwives for immunizations (primary preventions). They also educate at-risk people and perform screening through physical examinations, e.g., measuring the weight and height of toddlers, recorded in the *Posyandu* app (secondary preventions). If the cadres found an individual with a suspected illness, such as malnutrition or fever, they will contact the village midwife and then refer the case to the *Puskesmas*. The local government should emphasize a leadership and management practice to support and motivate the cadres to perform these tasks [65] to support the successful initiation of the PIS through the app and its integration with the national information system. Good leadership, communication, and coordination will engender a robust health information system in Indonesia [66].

## Conclusions

A hybrid approach is an essential and meaningful step in providing an intervention to fit the community’s needs with respect to the *Posyandu* services on data records and reporting. This 3-year hybrid approach with a user-centered design suggests the ideal phases in providing the basis to build a mobile app. The app can be created in a more user-friendly manner, can replace the CHWs’ old-fashioned book use, and can build “a bridge” between the community and national levels. The *Posyandu* app developed in this research promises to answer the existing national reporting system’s delay in practice. The cadres can contribute to the PIS by immediately inputting the data in real-time. Thus, it can automatically send reports faster to the *Puskesmas* and DHO.

This research also found that the cadres’ knowledge and skills displayed moderate and small improvements, respectively. Both are necessary for the cadres while performing *Posyandu* services in the field. Short dissemination of information followed by continuous monitoring, independent learning, and a user-friendly app will result in a satisfactory increase in the cadres’ knowledge and skills. The result may be equally satisfying for both the cadres who receive training and those who perform independent learning using the *Posyandu* mobile app. For further development, a new educational video that explains how to apply the app is recommended to replace direct or face-to-face disseminating information.

A limitation of this research is that it did not emphasize the need to increase the confidence and satisfaction of the CHWs when using the *Posyandu* app. If both aspects are considered in the future, it may extend the cadres’ knowledge and skills more effectively than the current research results.

## Supplementary Information

**Additional file 1: Supplemental Table 1.** End-user activities, the needs of the mobile app, and main features

**Additional file 2: Supplemental Table 2.** The use of *Posyandu* mHealth application by CHW

**Additional file 3: Supplemental Table 3.** Cadres and Village Midwives FGD Result on Posyandu Mobile App Development

**Additional file 4: Supplemental Table 4.** Advantages and Disadvantages Analysis of Posyandu Mobile Health Application

**Additional file 5: Supplemental Table 5.** Knowledge questionnaire about the *Posyandu* app

**Additional file 6: Supplemental Table 6**. *Posyandu* Application Use Observation Sheet

## Data Availability

The dataset supporting this article’s conclusions is not publicly available due to confidentiality but is available on reasonable request.

## Abbreviations

CHW: Community health worker/cadre
MCH: Mother–child health
PHC: Public health center
PIS: *Posyandu* Information System
FGD: Focus group discussion
IT: Information technology
SOP: Standard of procedure
DFD: Data flow diagram
