# Supplementary material for: Understanding mobile application development and implementation for monitoring Posyandu data in Indonesia: a 3-year hybrid action study to build “a bridge” from the community to the national scale
Source: BMC Public Health. 2021 May 31;21:1024. doi: 10.1186/s12889-021-11035-w (PMC8165997; doi:10.1186/s12889-021-11035-w)
Supplement: Supplementary file 1 — Additional file 1: Supplemental Table 1. End-user activities, the needs of the mobile app, and main features [file 12889_2021_11035_MOESM1_ESM.docx]

# Supplemental Table 1. End-user activities, the needs of the mobile app, and main features

| No | **Theme** | **Key insight and Noteworthy Quotes** |
| --- | --- | --- |
| 1 | End-user activities | |
|  | 1. CHWs’ activities | 1. The direct use of the CHWs’ notebook for MCH service   “The paper notebook can be used to document mothers’ attendance immediately. The data of five-year-old children are also written in the notebook; the three-year-old children’s data are also written. However, it is not in an orderly manner.”  “Out of all the data, the most awaited ones by the midwives for the next report are data recapitulation. For instance, the monthly data of how many infants gain weight, how many of them lose weight, how many come to the *Posyandu*, how many don’t, and the likes of that are the most awaited. The sum of how many comes is already a common thing.”   1. Delay in rewriting to the *Posyandu* information system (PIS) book   “After that, we have to write the names to the *Posyandu* information system book in an orderly manner.”  “written on a supplemental notebook first.”  “…rewritten (on the PIS) at home...”   1. PIS book is extremely hard to implement   “If the data is to be written immediately to the PIS as the mothers come, it will make my head blown (because it is) confusing.”  “(written) directly on the PIS is complicated because we already know their (names), their numbers, we already know.” |
|  | 1. Mothers’ activities | 1. Mothers who work will ask others to bring their children to the *Posyandu*:    - - babysitter      - grandmother      - neighbor   “For example, this (child), the child is taken care of by another person (because) the mother is working”  “Yes, nowadays, it is the grandmother who takes care of the baby, not the mother.”  “Or maybe the child is taken care of by the neighbor or other family members.” |
| 2 | The needs of mobile app | |
|  | 1. CHWs | 1. Monitoring infants, toddlers, and mother   “With the Posyandu mobile application, it is possible to check the development of children, examine pregnant mothers, immunization.”  “it’s easy because we don’t have to check this and that again, so we no longer feel shy (for asking repeatedly) because (the data) is now automatically (available)”   1. CHWs need a mobile app to report to *Puskesmas*   “It is like a tool, but it can be re-accessed, like an archive. Because we need it when *Puskesmas* requests (a report), sometimes it can be reaccessed.”  “the report is directly reported to the midwives, but it would be better if we can print (it). Therefore, we also have a copy, just in case…”  “yes, after the activities (the midwives can directly access it) …” |
|  | 1. Mothers | 1. Mothers need the mobile app to monitor their toddlers   “(We) need to know our child development so that we can monitor our by ourselves.”  “Yes, so we can privately look at it. So, we do not have to ask the CHW, ‘I want to see my son’s data, so how?’ It is just not practical.” |
| 3 | Main features | 1. Register and Login   “First, we click on the *Posyandu* app, then we register in it, after that we click it once more, then we are connected to our children’s data.”  “To format on, on that kind of tool, for toddlers, (it’s) not (supposed to be) alphabetically but sorted based on the date of birth. This is better. For example, if there is a newborn, s/he (her/his data) will be sorted automatically.”   1. 12-Month Reporting Format   “So, there should be a 12-month format in the reporting form to know whether the weight of the infant increases or decreases.”  “On the PIS, there is (a format) from January to December.”   1. Similar to the reporting form used in *Puskesmas*   “So, the format is similar to the PIS, but the mobile version is much more organized.”  “It is like a tool, but it can be re-accessed, like an archive. Because we need it when *Puskesmas* requests (a report), sometimes it can be reaccessed.”   1. Infant data input to be automatic data when the app is re-opened   “It would be better if the date can be automatically sorted so it will not be a hassle to find the infant’s date of birth like, “Oh, this infant was born on this date, this one on that date.” It would be easier if (the app) can automatically sort it alphabetically when we input the contact.”  “It would be even better if it (the app) can automatically sort the date (of birth) without the hassle of looking for (it).”   1. Child growth graph   “The satisfactory (growth graph) is the green one, right? This yellow one means that it is below (the green)...which is at least there is this graph for each child.”   1. Automatic alert of child growth   “…if it is possible, the increase and decrease status to be automatic. For example, last month (the child’s weight) was this much, this month it’s that much, so that number to automatically appear.” |

Description: Filling Instructions

Put a checkmark in the column provided in accordance with the steps/tasks undertaken by the cadre

1. : If it is not performed
2. : If it is performed with hesitation
3. : If it is performed with confidence
