# Supplementary material for: Understanding mobile application development and implementation for monitoring Posyandu data in Indonesia: a 3-year hybrid action study to build “a bridge” from the community to the national scale
Source: BMC Public Health. 2021 May 31;21:1024. doi: 10.1186/s12889-021-11035-w (PMC8165997; doi:10.1186/s12889-021-11035-w)
Supplement: Supplementary file 2 — Additional file 2: Supplemental Table 2. The use of Posyandu mHealth application by CHW [file 12889_2021_11035_MOESM2_ESM.docx]

# Supplemental Table 2. The use of *Posyandu* mHealth application by CHW

| **No** | **Theme** | **Key Insight and Noteworthy Quotes** |
| --- | --- | --- |
| 1 | Toddler Data Input | 1. Toddler body weight measurement   “Monthly weight data is automatically noted.”  “So to know if the children’s weight is increased or decreased, (it) can be seen on the graph.”   1. Toddler body height measurement time   “We do not measure the height monthly, only once several months.”  “Weight is measured every month, but height can be skipped because it is not measured monthly.”   1. Toddler body height measurement according to WHO   “The measurement method is by lying (the child) down or standing up.”  “Measuring the height should be available on the application. It is called length for infants and height for toddlers.” |
| 2 | Toddler Data Display | 1. Toddler data can be accessed anytime   “So, every month is like the last month. For example, an infant is measured in January, in February s/he is measured as well, then the February report will appear when we click the child’s name.”  “If (we) want to know whether the child’s weight is increased (or) decreased, (the data) can be seen on the application anytime.”   1. Toddler measurement result display   “So, when all the data has a result, will the graph appear?”  “Every month during *Posyandu*, when filling in (the data on) children’s wight, the increased (or) decreased display is directly seen.”   1. Parents can access toddler data   “Sometimes the parents are like, ‘ma’am does my baby’s weight increase or not?’, then we can show the data in the application.”  “Sometimes there are parents who want to know the increase (or) decrease in their children’s weight, (when this happens, we) can show them directly on the application.” |
| 3 | *Posyandu* Mobile App Components | - 1. *Posyandu* mobile app menu   “There are infants’ names, the ups and downs of toddler data, pregnant mothers, breastfeeding mothers, etc.”  “Body length and body height of children also have to be available.”   - 1. Online report   “We want it to be like (...) online reporting, so we do not need to measure the number of decreases.”  “All data, when they have been inputted, (they) directly go online, we just have to log in without having to wait long, and the data is delivered.” |
| 4 | Benefits of *Posyandu* Mobile app | 1. *Posyandu* reporting and recording is easier   “Directly input the data, input the data so that the report will be directly submitted to *Puskesmas.*”  “(I) think obviously (we are) helped during the reporting process. We don’t have to calculate again, how much is the increase in the children’s weight, how much is the decrease, like that.”   1. Ease cadres’ duties in *Posyandu*   “So, the recording division does not need to note manually to the SIP book, but the input can be done through the application.”  “Usually after *Posyandu* (we), make the report at home, but when we have this (the Posyandu mHealth), we don’t have to calculate anymore.” |
| 5 | Cadres’ obstacle in using the *Posyandu* mobile app | 1. Confused/need to adapt   “A while ago, some data was successfully stored, but some were unsuccessful.”  “Just so that we can (use it), ma’am, when the tool is available, teach us again, guide (us) in inputting the infants’ data.”  “Study like school.”   1. Unsupportive *Posyandu* situation   “During *Posyandu* working day, it will remain crowded so that the data entry will be done after the end.”  “If (we) directly input children’s data to the application, there must be queues.”  “If the cadres are in full formation, there is no problem. The problem is when we don’t have enough cadres.” |
| 6 | Learning process | 1. Cadres’ knowledge of the *Posyandu* mobile app   “We were trained; one post held the tool. We received training to input the data.”  “I have tried it earlier but still don’t understand.”   1. Cadres’ skills on the use of *Posyandu* mobile app   “We think that we can use it because we are used to using and playing with a mobile phone (Android). However, before that, the application should be made available first (on Google Play).”  “Because (it) has not been applied, (we are) still kind of guessing, (we) still need practice.”   1. Cadres need more training   “When it is time to learn, all of us should be gathered like training.”  “We (need to be) guided, every *Posyandu* (need to) have the tool, and we (need to be) taught to input the infants’ data.” |
| 7 | *Posyandu* mobile app guidebook | 1. Significance of *Posyandu* mobile app guidebook   “A guidebook is necessary because we often forget things.”  “So that the tool can exist (be used), it should have a guidebook.”   1. Guidebook format   “A small format is appropriate” (while the woman pointed at an A5 paper)  “The form is like a pocketbook where there are procedures to log in and the steps.”   1. Guidebook size   “The letters should not be too small.”  “The size just needs to be like a pocketbook.”   1. Guidebook writing style   “The writing should be black and white.”  “The writing needs to be in uppercase.”  “Just adjust (make it appropriate) it, ma’am.”   1. Images in the guidebook   “If the writing is black and white, the images should be colored.”  “The images should be in color, so they are clear, the screenshot also (should be) in color.” |
| 8 | Information in the guidebook | 1. Instruction   “In the manual guidebook, there should be a set of instructions and screenshots.”  “The steps are described, and there are descriptions of the parts next to them as well as images.”   1. How to register an account/log in   “Such as how to do login and accompanied by a screenshot beside.”  “The registration will use mobile phone numbers, so mobile phone numbers need to be memorized.”  c. How to input toddler data  “For example, if we want to input toddler data, we should click this if we want to add it.”  “During Posyandu, (we) need to input the date once. After that, (we) just input the toddler data.”  d. How to input pregnant mother data  “Usually, pregnant mother data has a record on the number of children.”  ”So other than the name of the pregnant mothers, the name of the husbands need to be inputted as well” |
| 9 | Cadres’ hopes | 1. Tablet/mobile phone provision   “Yes, if there are three *Posyandu* in a village, then there should be three (tablet/mobile phone) in a village.”  “Ideally, there should be one tablet for one *Posyandu*”   1. Use of application in *Posyandu*   “If using the application if possible, then so be it, (I) cannot wait to use it.”  “(We are) grateful for the help, hopefully (it is) realized soon.” |
| 10 | Cadres’ concerns | Internet quota availability  “Well, *Posyandu* does not have the budget, I asked the villagers about the internet quota fee, and they already shook their heads.”  “Hopefully, next year, it is included in the village budget.” |

Description: Filling Instructions

Put a checkmark in the column provided in accordance with the steps/tasks undertaken by the cadre

1. : If it is not performed
2. : If it is performed with hesitation
3. : If it is performed with confidence
