# Supplementary material for: Understanding mobile application development and implementation for monitoring Posyandu data in Indonesia: a 3-year hybrid action study to build “a bridge” from the community to the national scale
Source: BMC Public Health. 2021 May 31;21:1024. doi: 10.1186/s12889-021-11035-w (PMC8165997; doi:10.1186/s12889-021-11035-w)
Supplement: Supplementary file 3 — Additional file 3: Supplemental Table 3. Cadres and Village Midwives FGD Result on Posyandu Mobile App Development [file 12889_2021_11035_MOESM3_ESM.docx]

# Supplemental Table 3. Cadres and Village Midwives FGD Result on Posyandu Mobile App Development

| No. | Theme | Key Insight and Noteworthy Quotes |
| --- | --- | --- |
| 1. | Identity | Village name correction in the editing menu  “Here, the name of my village in this application is wrong.  “Warung Kadu (correct village name), still Warung Kidul (incorrect village name).” (Cadre)  “Sawah Kulon (correct village name) is still written as Pasawahan Kulon (incorrect village name).” (Midwife)  “Warung Kadu (correct village name) is also still Warung Kidul (incorrect village name). That’s an incorrect village name.” (Midwife) |
| 2. | Account | - 1. Account owner photo   “Ma’am, can we put our photo so that there will not only be our name (displayed) in it? Even Google mail has the owner’s photo. This will result in a higher sense of belonging.” (Cadre)   - 1. Alternative password   “Oh, I forgot the password. It is not possible to login. It would be great if an alternative password exists.” (Midwife)   - 1. Individual account for Village Midwife   “That is, in the future, it would be great if there is access for the village midwives and not only for the cadres. So, it will not be a hassle to input (the data) one by one. Now, there is only 5 *Posyandu* in one village in different locations, and one village midwife handles even 13 *Posyandu.*” (Midwife) |
| 3. | Website | Web creation  “We hope that there will be a web to monitor at a larger scale for the midwives. The web can make it easier to read the report.” (Midwife)  “Yes, it is expected (that we can) monitor in a larger (scale). Sometimes it’s tiring to see (the data). It would be better if there is a web because it would just like WhatsApp”. (Cadre)  “Well, a web for the report would be good to access.” (Cadre) |
| 4. | New menu | Pregnancy age automatic calculation  “It is hoped that after the input of the first day of the last period, the pregnancy age will be automatically calculated in the next examination schedule.” (Midwife) |

Description: Filling Instructions

Put a checkmark in the column provided in accordance with the steps/tasks undertaken by the cadre

1. : If it is not performed
2. : If it is performed with hesitation
3. : If it is performed with confidence
