# Supplementary material for: Understanding mobile application development and implementation for monitoring Posyandu data in Indonesia: a 3-year hybrid action study to build “a bridge” from the community to the national scale
Source: BMC Public Health. 2021 May 31;21:1024. doi: 10.1186/s12889-021-11035-w (PMC8165997; doi:10.1186/s12889-021-11035-w)
Supplement: Supplementary file 4 — Additional file 4: Supplemental Table 4. Advantages and Disadvantages Analysis of Posyandu Mobile Health Application [file 12889_2021_11035_MOESM4_ESM.docx]

# Supplemental Table 4. Advantages and Disadvantages Analysis of Posyandu Mobile Health Application

| No | Analysis Results | Advantages (A)/Disadvantages (D) |
| --- | --- | --- |
| 1 | User | |
|  | 1. Receipt | A: Cadre’s approval of the application  “If this my phone, I’ll try to use it.” (Cadre)  “If a cadre is to ask, ‘what is the date of birth of this child?’ I would say, ‘We have that data now on iPosyandu, just install it and access (the data), so there is no need to ask again.’” (Cadre)  “I don’t think there should be any training because this is easy. Just do socialization once and (they/we) will be able to learn autonomously.” (Midwife)  “Well, that’s how millennials are, especially this application is easy to use.” (Midwife) |
|  | 1. Problem Solving | A: Problem solution by the cadres when facing difficulty.  “If there is no quota, then just buy some.” (Cadre)  “Just use tethering connection from me, easy.” (Midwife)  “Um… how much would the budget for monthly quota be and how much for every activity. (Midwife)  “Yes, that’s how I would do it. Last time, there were three of us without internet quota, and then I said, ‘here I have immunization money, let’s take ten thousand rupiahs each.’” (Cadre) |
|  | 1. Skill | A: Cadres’ skill in operating the *Posyandu* Mobile app  “This is very easy to use. One socialization and (we/they) will be able to use it. Just use it often, then it will be fine.” (Midwife)  “Oh right, just use one-hand (feature). While weighing, while inputting.” (Midwife)  “(We) are already (do it) smoothly, ma’am, so we are not afraid.” (Cadre)  “The implementation (takes place) on table 1 and table 3.” (Cadre) |
|  | 1. Resistance | D: Time-consuming because there is a reluctance on the part of some cadres to change into digital-system-based services  “Sometimes (we) cannot do it directly, ma’am, because it’s a lot of hassle; so many patients come at the same time.” (Cadre)  “When people are coming, all of them. Hectic.” (Cadre)  “So (we are) jealous if we do it alone.” (Cadre) |
| 2 | Organization | |
|  | 1. Policy | A: Presence of a village midwife leadership as the direct supervisor of Posyandu activities.  “There will be cadre meetings, and in these cadre meetings, it will be evaluated.” (Midwife)  “Then, we will also choose to be visited for networking.” (Midwife)  “Agree. Networking visit will be able to identify which has problems.” (Midwife)  “Well, with us having the cadres’ accounts, we will be able to do the monitoring.” (Midwife)  “We will be available; we are there during *Posyandu.* Therefore, we can see which cadres are lazy.” (Midwife)  “I agree, cadres must always be accompanied.” (Midwife)  “Don’t forget about distributing work with us. Village midwives are the one with control over distributing work in table 1 and table 3, so nobody gets jealous. Jealous about work.” (Midwife)  “Delegate um… for example, um… if the goal is 60 minutes, (then) it’s 30-30, or 20-20-20 so that everyone can (do it), ma’am.” (Cadre)  “Well, if it is done, it is to be distributed.” (Cadre) |
|  | 1. Organizational Support | Cadres’ facility in the implementation of the *Posyandu* mobile app is still necessary  “(I) would like to have one specific Android (device) for *Posyandu*. A while ago, Dr. Fedri had met the *Camat* (official village leader), but the *Camat* is replaced.” (Cadre)  “Need quota” (Cadre)  “Well, what I want is, for instance, um… if um… I (use) my own quota, right, that must be the case. If the midwife said it instead, they would listen, but they wouldn't listen if I said it.” (Cadre) |
|  | 1. Standard Operating Procedure (SOP) | A: Positive response in the advocacy of the government  “A while ago, Dr. Fedri had met the *Camat* (official village leader).” (Cadre)  “The need for quota proposed to the village office.” (Cadre)  D:  Double work burden between manual and digital tasks because no SOP regulates the implementation of the *Posyandu* mobile app  “Also, since this is maybe still a transition, the cadres still do double tasks even though it would be easier to use the application.” (Midwife)  “So here’s the thing, there are maybe still so many visitors, but (what happens is) still the app is being used, the PIS book is being used, and the personal notebook is still being used.” (Cadre)  “In my case, last time, because the SIP book is still being used, so we still write on the PIS book, um… the uploading process is also still done. So there are double tasks.”(Cadre) |
| 3 | Technology | |
|  | - 1. Application/Software | |
|  | 1. Accuracy | A: Appropriate and correct use of the application  “There will be no such thing as missing data, even from ten years ago.” (Midwife) |
|  | 1. Facility | A: User-friendliness of the application when being operated by the cadres  “This is very easy to use. One socialization and (we/they) will be able to use it. Just use it often, then it will be fine. “(Midwife)  “During the weighing process, (we) just input the data, apparently easy.” (Cadre)  “Weighing and inputting at the same time” (Cadre)  “Especially this application is easy to use.” (Midwife)  “Oh right, just use one-hand (feature). While weighing, while inputting”. (Midwife)  “Due to high interaction with gadgets, this is easy to use.” (Midwife) |
|  | 1. Availability | A: Availability of the application on Google Play to be used or operated  “If a cadre is to ask, ‘what is the date of birth of this child?’ I would say, ‘We have that data now on iPosyandu, just install it and access (the data), so there is no need to ask again”. (Cadre)  “Sometimes the midwives also ask the date of birth is, when the year of birth is.” (Cadre)  “Yes, being used directly during examination is faster and more orderly.” (Midwife) |
|  | 1. Relevance | A: Conformity of the application menu with the needs of the cadres or as planned by the government  “This can be carried easily; while the SIP is a book, this one is direct.” (Cadre)  “The target is very clearly shown.” (Cadre)  “Praise be to God, this is application is complete. Even the immunization is complete.” (Midwife)  “Yes, it’s very good; practical. Just print the report.” (Midwife)  “Especially because this application is following the PIS format from the government.” (Midwife) |
|  | 1. Punctuality | A: Real-time condition of the application to display the information or examination result  “Whenever there is a question, (we) are ready to answer ma’am.” (Cadre)  “The system is as usual, maybe because the signal is good, so it is directly inputted.” (Cadre)  “Yes, being used directly during examination is faster and more orderly.” (Midwife)  D: In the region with low network coverage, an offline version of the application is needed for data entry, which will be submitted after the network appears  “Yes, ma’am, so when we upload (the data), it would be better if (the application) doesn’t use (internet connection), so just offline. When we are done, then we can upload the data.”  “So, when online (the application) can update automatically.”  “Yes, it goes back to the loading (page). This is the one in the urban area, (the situation) is worse for those in the village are like Selaawi.” (Cadre) |
|  | 1. Challenge | D: Application bugs need to be fixed  “Sometimes it keeps loading.” (Cadre)  “When we input the data, there’s no problem, then we upload (the data) Bla bla bla… then it gets slow.” (Cadre) |
|  | - 1. Hardware | |
|  | 1. Network | D: Unstable network for some providers/carriers  “The challenge is that sometimes the loading is hindering (the process). The network. Hahaha…” (Cadre)  “And sometimes it keeps loading.” (Cadre)  “Yes, loading.” (Cadre)  “When we input the data, there’s no problem, then we upload (the data) bla bla bla… then it gets slow.” (Cadre)  “Yes, me too, same. Sometimes it keeps loading.” (Cadre) |
|  | 1. Handphone | D: Some versions of android are not compatible with the application  “But the challenge is the error on the mobile phone .” (Cadre)  “The biggest challenge is that it takes long to load. Also, when they don’t have an Android mobile phone, we cannot do anything.” (Cadre)  “Sometimes the Android version is incompatible. So (the application) is limited to new Androids. So, those who have (older) Androids are disappointed because (their devices) are outdated..” (Cadre)  “The Android was incompatible. If it’s possible, ma’am, make (the application) compatible for lower Android versions..” (Cadre) |
|  | 1. Quota | D: Some cadres do not have an internet quota  “The quota runs out.” (Cadre)  “My cadres complain about quota.” (Midwife) |
| 4. | Environment | D: The situation that is not conducive (queueing issue) during *Posyandu*’s business time  “(They) don’t want to queue. If we didn’t do what we did, (it would be) chaotic.” (Cadre)  “Want to go quickly.” (Cadre)  “Usually very crowded...” (Cadre) |

Description: Filling Instructions

Put a checkmark in the column provided in accordance with the steps/tasks undertaken by the cadre

1. : If it is not performed
2. : If it is performed with hesitation
3. : If it is performed with confidence
