# Supplementary material for: Understanding mobile application development and implementation for monitoring Posyandu data in Indonesia: a 3-year hybrid action study to build “a bridge” from the community to the national scale
Source: BMC Public Health. 2021 May 31;21:1024. doi: 10.1186/s12889-021-11035-w (PMC8165997; doi:10.1186/s12889-021-11035-w)
Supplement: Supplementary file 5 — Additional file 5: Supplemental Table 5. Knowledge questionnaire about the Posyandu app [file 12889_2021_11035_MOESM5_ESM.docx]

# Supplemental Table 5. Knowledge questionnaire about the *Posyandu* app

| **No** | **Question** | **Answer** | |
| --- | --- | --- | --- |
|  |  | **Correct** | **Incorrect** |
| **Account management** | | | |
| 1. | Before using the *Posyandu* application, the cadres must register their account first |  |  |
| 2. | The option to register a *Posyandu* app account is available as a cadre and as a parent |  |  |
| 3. | The cadre biography must be filled in the format available when registering an account on the *Posyandu* application |  |  |
| 4. | Once an account is registered, the cadres can simply log in if they are going to use the *Posyandu* application |  |  |
| 5. | An internet connection is required to be available in using the *Posyandu* application, |  |  |
| ***Posyandu* application advantages** | | | |
| 6. | The *Posyandu* application helps the cadres in carrying out *Posyandu* activities, especially for recording and reporting |  |  |
| 7. | The *Posyandu* application contains health information for infants and toddlers and pregnant women |  |  |
| 8. | In addition to the cadres, the *Posyandu* application can also be used by mothers with infants and toddlers |  |  |
| **Pregnant women data** | | | |
| 9. | Pregnant women data on the application contains the mother’s identity and weight of the mother pregnancy |  |  |
| 10. | To make it easier for the cadres to find data on pregnant women who are already in the application, the names of pregnant women shall be sorted in an alphabetical order |  |  |
| 11. | Data of pregnant women who are not yet included in the *Posyandu* application can be added to the application |  |  |
| **Pregnant women inspection results** | | | |
| 12. | Data on pregnant women contains the results of examinations of pregnant women, such as gestational age, weight of pregnant women, and height of pregnant women |  |  |
| 13. | Displaying the results of examinations of pregnant women in the application can be done at any time, outside the *Posyandu* schedule |  |  |
| 14. | The results of the examination of pregnant women in the previous months can be seen in the data menu of the results of pregnant women examination by date |  |  |
| 15. | Prescription of iron supplement pills needs to be included in the examination results on the *Posyandu* application |  |  |
| **Toddler Data** | | | |
| 16. | All infants and toddlers in the *Posyandu* area must be included in the *Posyandu* application data |  |  |
| 17. | To children's data on the infants and toddlers’ data menu, it is required to include the parents’ name |  |  |
| 18. | Toddler data recording on the *Posyandu* application is carried out until the child is more than 5 years old |  |  |
| 19. | Data on infants and toddlers are inputted in the same menu as pregnant women data |  |  |
| **Inspection Results on Toddlers** | | | |
| 20. | In addition to the children’s weight, the children’s height is also included in the infant and toddler data in the application |  |  |
| 21. | Exclusive breastfeeding data and administration of vitamin A are included in the results of examinations for infants and toddlers |  |  |
| 22. | Weight gain for infants and toddlers can be seen on the weight gain graph in the application |  |  |
| ***Posyandu* Information System** | | | |
| 23. | *Posyandu* Information System (PIS) has 6 formats available in the application |  |  |
| 24. | The PIS format is filled at the beginning and end of the year, not every month |  |  |
| 25. | After completing the application’s *Posyandu* data, it can be reported immediately |  |  |

Description: Filling Instructions

Put a checkmark in the column provided in accordance with the steps/tasks undertaken by the cadre

1. : If it is not performed
2. : If it is performed with hesitation
3. : If it is performed with confidence
