# Supplementary material for: Understanding mobile application development and implementation for monitoring Posyandu data in Indonesia: a 3-year hybrid action study to build “a bridge” from the community to the national scale
Source: BMC Public Health. 2021 May 31;21:1024. doi: 10.1186/s12889-021-11035-w (PMC8165997; doi:10.1186/s12889-021-11035-w)
Supplement: Supplementary file 6 — Additional file 6: Supplemental Table 6. Posyandu Application Use Observation Sheet [file 12889_2021_11035_MOESM6_ESM.docx]

Supplemental Table 6. *Posyandu* Application Use Observation Sheet

| **No.** | **Activities** | **0** | **1** | **2** |
| --- | --- | --- | --- | --- |
| **1** | **Registering account:** |  |  |  |
|  | - Press the ‘register’ button on the home screen display |  |  |  |
|  | - Press the ‘register as a cadre’ button |  |  |  |
|  | - Fill in Posyandu data that includes Province, Regency / City, District / Sub-district, *Puskesmas* and *Posyandu* |  |  |  |
|  | - Fill in personal account data in the available fields (full name, place of birth, date of birth, and address) |  |  |  |
|  | - Fill in the data account by inputting the phone number and password |  |  |  |
|  | - Confirm password with the same password |  |  |  |
|  | - Press the ‘send registration’ button on the screen |  |  |  |
| **2** | **Log in to the application** |  |  |  |
|  | - Press the ‘login’ button on the home screen |  |  |  |
|  | - Fill in the telephone number used when registering an account |  |  |  |
|  | - Fill in the password used when registering an account |  |  |  |
|  | - Press the ‘login’ button on the screen |  |  |  |
| **3** | **Fill in toddler data** |  |  |  |
|  | - Press the ‘infant and toddler’ menu on the home screen |  |  |  |
|  | - Press the ‘+’ button on the lower-right corner of the screen (infant and toddler data fields) |  |  |  |
|  | - Fill in the data of infants and toddlers in the infant and toddler register column (child’s name, date of birth, child to, sex, NIK/Identity Number, birth weight in kilograms, father’s name, mother’s name, mother’s NIK/Identity Number, telephone name, address, RT, RW) |  |  |  |
|  | - Press the save button |  |  |  |
| **4** | **Displays data on toddlers** |  |  |  |
|  | - Press the ‘infant and toddler’ button on the home screen |  |  |  |
|  | - Choose the desired name of the infant/toddler |  |  |  |
|  | - Press the infant/toddler name in the infant and toddler register column in the *Posyandu* area |  |  |  |
| **5** | **Looking for toddler data** |  |  |  |
|  | - Press the ‘search’ button on the top left corner of the infant and toddler register column |  |  |  |
|  | - Write the name of the infant/toddler you are looking for in the search field |  |  |  |
|  | - Press the search button on the lower-right corner of the keyboard screen |  |  |  |
| **6** | **Fill in toddler examination data** |  |  |  |
|  | - Press the ‘Posyandu’ menu |  |  |  |
|  | - Press the plus button (+) on the lower-right corner of the screen in the infant and toddler data column |  |  |  |
|  | - Fill in infant and toddler data on the infant and toddler register column (infant/toddler name, check date, weight in kilograms, height in centimeters, measurement method, immunization, early breastfeeding initiative, exclusive breastfeeding, vitamin A for February/August, MCH book, PMT, and diarrhea) |  |  |  |
|  | - Press the ‘save’ button on the screen to save the data |  |  |  |
| **7** | **Display toddler data** |  |  |  |
|  | - Press the ‘*Posyandu*’ menu |  |  |  |
|  | - Press the inspection date |  |  |  |
|  | - Press the name of the infant/toddler who wants to know the data of the results of the examination |  |  |  |
| **8** | **Fill in the data of pregnant women** |  |  |  |
|  | - Press the ‘pregnant woman’ menu on the home screen |  |  |  |
|  | - Press the plus button (+) on the lower-right corner of the screen in the data column of pregnant women |  |  |  |
|  | - Fill in the data of pregnant women (name of pregnant women, husband’s name, Identity Number of mother and husband, date of birth of mother, address, telephone number, and weight during pregnancy) |  |  |  |
|  | - Press the ‘save’ button on the screen to save the data |  |  |  |
| **9** | **Display pregnant women data** |  |  |  |
|  | - Press the menu of pregnant women on the home screen |  |  |  |
|  | - Press the name of the pregnant woman available in the data column of pregnant women |  |  |  |
| **10** | **Looking for data on the results of examinations of pregnant women** |  |  |  |
|  | - Press the search button on the upper-left corner of the home screen |  |  |  |
|  | - Fill in the name of the desired pregnant woman |  |  |  |
|  | - Press the search button on the lower-left corner of the keyboard screen |  |  |  |
| 11 | **Log out of the application** |  |  |  |
|  | - Press the ‘log out’ button on the upper-right corner of the home screen |  |  |  |

Description: Filling Instructions

Put a checkmark in the column provided in accordance with the steps/tasks undertaken by the cadre

1. : If it is not performed
2. : If it is performed with hesitation
3. : If it is performed with confidence
